# Multi-Level Processes and Retina–Brain Pathways of Photic Regulation of Mood

**DOI:** 10.3390/jcm11020448

**Published:** 2022-01-16

**Authors:** Julia Maruani, Pierre A. Geoffroy

**Affiliations:** 1 Département de Psychiatrie et d’Addictologie, AP-HP, GHU Paris Nord, DMU Neurosciences, Hôpital Bichat—Claude Bernard, F-75018 Paris, France; 2 NeuroDiderot, INSERM U1141, Université de Paris, F-75019 Paris, France; 3 CNRS UPR 3212, Institute for Cellular and Integrative Neurosciences, 5 rue Blaise Pascal, F-67000 Strasbourg, France; 4 GHU Paris—Psychiatry & Neurosciences, 1 Rue Cabanis, F-75014 Paris, France

**Keywords:** light, light therapy, mood, suprachiasmatic nuclei (SCN)

## Abstract

Light exerts powerful biological effects on mood regulation. Whereas the source of photic information affecting mood is well established at least via intrinsically photosensitive retinal ganglion cells (ipRGCs) secreting the melanopsin photopigment, the precise circuits that mediate the impact of light on depressive behaviors are not well understood. This review proposes two distinct retina–brain pathways of light effects on mood: (i) a suprachiasmatic nucleus (SCN)-dependent pathway with light effect on mood via the synchronization of biological rhythms, and (ii) a SCN-independent pathway with light effects on mood through modulation of the homeostatic process of sleep, alertness and emotion regulation: (1) light directly inhibits brain areas promoting sleep such as the ventrolateral preoptic nucleus (VLPO), and activates numerous brain areas involved in alertness such as, monoaminergic areas, thalamic regions and hypothalamic regions including orexin areas; (2) moreover, light seems to modulate mood through orexin-, serotonin- and dopamine-dependent pathways; (3) in addition, light activates brain emotional processing areas including the amygdala, the nucleus accumbens, the perihabenular nucleus, the left hippocampus and pathways such as the retina–ventral lateral geniculate nucleus and intergeniculate leaflet–lateral habenula pathway. This work synthetizes new insights into the neural basis required for light influence mood

## 1. Introduction

Light therapy (LT) is an old treatment used since antiquity with sun therapies applied in ancient Chinese, Hindu, and Egyptian medicine since the 15th century BC [1]. Humans from all latitudes have always worshiped the sun, which has been repeatedly and all around the world considered as a god. Wong Tai, more than 4700 BC, was one of the first to describe a real link between seasonal changes in light and variations in mood [1]. Later, the very first medical descriptions reporting light efficacy on mood were made by Hippocrates who wrote on the interrelation between seasonal climates and mood, and by Aretaeus de cappadocia who for instance wrote that “lethargic are to be laid in the light, and exposed to the rays of the sun for the disease is gloom” [1]. Heliotherapy, has been the longest used form of LT and the only one until the mid-nineteenth century [1]. Then with the invention of the electric light bulb and the progress of medicine, heliotherapy shifted to ultra-violet (UV) phototherapy and to LT, which is an UV filtered light, still used nowadays [1]. The recent rebirth of LT comes from neurosciences, with two key findings in 1979 and 1980. The first discovery was that phase shifts of circadian rhythms can have an antidepressant effect [2] and the second that light suppresses melatonin secretion and so can impact circadian rhythms [3]. The link between these two findings was quickly made and the antidepressant effect of LT by inducing a phase advance and/or alignment of circadian rhythms was confirmed by Kripke et al. [4,5] and Lewy et al. [6]. Nevertheless, it has been then proposed that LT effects might be also associated with mechanisms independent of effects on the biological clock. It was indeed suggested by past observations that light effects on circadian rhythm did not correlate well with antidepressant efficacy, as confirmed for instance by an observed efficacy even when LT was administered at midday [7,8]. These direct and indirect effects on mood may explain why light exerts a strong and rapid effect on depression [7,9]. LT mainly came to the attention of clinicians with the publication by Rosenthal et al. of a case-series of patients successfully treated for seasonal affective disorder (SAD) in 1984 [10]. Several studies and randomized controlled trials followed and confirmed that LT is more efficient than placebo in subjects with both unipolar depression [11] and bipolar depression [12] with seasonal patterns. Moreover, LT antidepressant effects was also observed in non-seasonal depression [11,13]. LT can be used as a monotherapy, in combination to antidepressants, or as an add-on/augmentation strategy for all subtypes of depression [14,15], increasing the response to antidepressants in non-responder patients [16]. In line with this, a recent meta-analysis confirmed the need to change practices and proposed to use LT as a first line treatment [17]. Authors also recommended the first-line combination treatment in order to maximize patients’ response rates rather than using LT as a second line treatment [17]. Of note, LT should not be considered by physicians only as the cornerstone treatment of SAD [10], but also as an efficient antidepressant strategy in non-seasonal depressions, with effect sizes equivalent to selective serotonin reuptake inhibitors (SSRIs) [11,13].

A better understanding of the mood regulation by light is important to fully use and personalize LT in individuals suffering from depression. LT benefited from major findings made over past decades. The recent discovery of melanopsin, a photopigment maximally sensitive to blue wavelengths and expressed in a subset of intrinsically photosensitive retinal ganglion cells (ipRGCs), allowed for a better understanding of light effects and for the identification of the neural substrates of the photic regulation of mood [18,19]. Nevertheless, the precise circuits that mediate the impact of light on depressive behaviors are not well understood and sparse evidence accumulate with no links made between all these researches. Understanding these neural bases for mood regulation by light constitutes a key step to personalize strategies and develop new chronotherapeutics in mood disorders. Therefore, in this extensive narrative review, we decided to synthetize new insights regarding how light may influence mood with particular emphasis on recent findings of light effects on mood through the new identified SCN-independent pathways, i.e., not solely depending on the synchronization of the biological clock.

## 2. Materials and Methods

### 2.1. Mood Definitions

Emotions should not be confounded with mood disorders and need to be clearly defined to better understand the scientific literature and studies’ results about mood regulation by light. Emotions are defined by brief responses characterized by a physiological arousal that are triggered by a stimulus to drive an adapted behavior [20,21,22] and are characterized by two quantifiable features: (i) the intensity of the response and (ii) the valence. Emotional valence is the subjective value assigned to sensory stimulus, which determines subsequent behavior. Positive valence leads to approach behaviors while negative valence leads to defensive and avoidance behaviors [23]. Stimuli can be external via our senses or internal. The perception of these stimuli will directly influence the behavioral response.

Mood can be considered as an emotion related-process with longer experiences associated with both positive and negative affect. Some authors suggest that emotion and mood are distinct entities, but the differences between emotion and mood lead to conflicting results and suggestions [24]. We thus decided to consider and define mood disorders, which are entities with clear definitions and validated criteria distinct from emotions [25].

Bipolar disorder (BD) and major depressive disorder (MDD) are described by the American Psychiatric Association’s Diagnostic and Statistical Manual of Mental Disorders (DSM-5)—the most used psychiatric disorder classification—as two groups of brain disorders that cause extreme fluctuation in a person’s mood, energy, and ability to function [25]. BD is a severe chronic mood disorder characterized by the recurrence of depressive and manic episodes. BD is defined according to the longitudinal course, which is often characterized by the presence of subthreshold symptoms. To be diagnosing a manic episode, the elevated, expansive, or irritable mood must last for at least one week and be present most of the day, nearly every day. To define a hypomanic episode, symptoms must last at least four consecutive days and be present most of the day, almost every day. During this period, three or more of the following symptoms must be present and represent a significant change from usual behavior: elevated, expansive, or irritable mood, decreased need for sleep, inflated self-esteem or grandiosity, increased talkativeness, racing thoughts, distracted easily, increase in goal-directed activity or psychomotor agitation, engaging in activities that hold the potential for painful consequences, e.g., unrestrained buying sprees.

The depressive side of BD is characterized by a major depressive episode resulting in depressed mood or loss of interest or pleasure in life. The DSM-5 states that a person must experience five or more of the following symptoms in two weeks to be diagnosed with a major depressive episode: depressed mood most of the day, nearly every day, loss of interest or pleasure in all, or almost all, activities, significant weight loss or decrease or increase in appetite, insomnia or hypersomnia, engaging in purposeless movements, such as pacing the room, fatigue or loss of energy, feelings of worthlessness or guilt, diminished ability to think or concentrate, or indecisiveness, recurrent thoughts of death, recurrent suicidal ideation without a specific plan, or a suicide attempt [25].

MDD are characterized by the recurrence of depressive mood symptoms with at least 5 symptoms during the same two weeks period that are a change from previous functioning among: depressed mood, loss of interest/pleasure, weight loss or gain, insomnia or hypersomnia, psychomotor agitation or retardation, fatigue, decreased concentration, thoughts of death/suicide, feeling worthless or excessive/inappropriate guilt [25].

Disruption emotional responses are a transnosographic dimension which can affect patients with BD and MDD. Emotional processing is even among the most affected dimensions in bipolar disorders (BD), but is excluded from the definition criteria. Emotional reactivity referred to emotion response intensity and emotion response threshold. Higher emotion reactivity is described during both mood episodes and periods of remission in BD. Although considered in remitted phases, BD patients experience more frequent and intense emotions in response to environmental conditions, compared to healthy subjects, which leads to mood instability [26].

### 2.2. Literature Search and Strategy

We aimed to consider papers examining neuronal circuits involved in light mediated effect on mood. Therefore, we conducted a narrative review using PubMed and Google Scholar databases up to June 2021, using the following keywords combination: (“depression” or “bipolar disorder” or “unipolar disorder” or “seasonal affective disorder”) and (“light” or “bright light” or “blue light”) and (“light therapy” or “ bright light therapy” or “phototherapy” or dim light therapy) and (“light retina brain pathway” or “light influence on mood” or “effect of light on mood” or “SCN effect of light on mood ” or “circadian effect of light on mood ” or “non circadian effect of light on mood ”).

### 2.3. Study Selection

**Figure 1:**
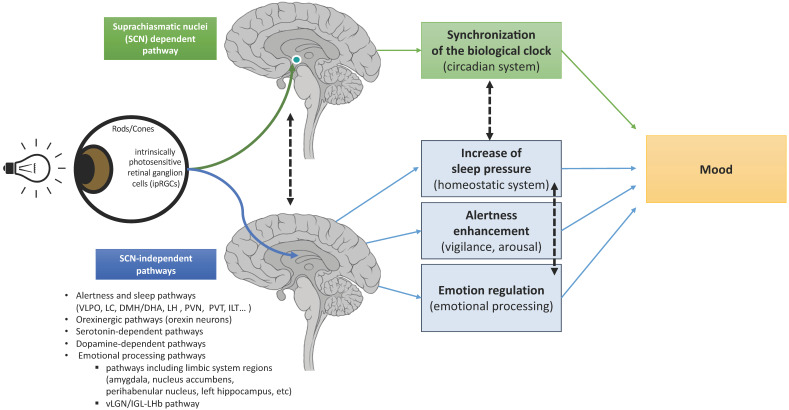
Multi-level processes and retina–brain pathways of photic regulation of mood.

Two authors (J.M., P.A.G.) reviewed the title and abstract of identified publications in order to identify eligible studies. J.M. and P.A.G. independently and then jointly selected studies for detailed extraction of information, mostly based on the full text. We then decided to divide literature results in two main sections for light actions on mood and retina–brain pathways: (i) light effects on mood through an SCN-dependent pathway and (ii) light effects on mood through SCN-independent pathways (Figure 1). The two sections summarize the information reviewed about the different pathways involved in light actions on mood. To better unravel this complex and multi-levels scientific literature, the second section about SCN-independent pathways will be subdivided in 4 sub-sections: (i) sleep and alertness pathways; (ii) orexinergic pathways, (iii) monoamine-dependent pathways, and (iv) emotional processing pathways.

There are two distinct retina–brain pathways for effect of light on mood: (i) a SCN-dependent pathway activated by the intrinsically photosensitive retinal ganglion cells (ipRGCs) synthetizing the melanopsin photopigment in response to light. SCN perceive and encode light information, then synchronize biological rhythms and project light information to many areas including: the sleep-inducing ventrolateral preoptic (VLPO) nucleus, structures involved in wake and appetite—such as the lateral hypothalamus LHA-, areas directly involved in mood regulation—such as the dorsal raphe-, the corticosteroid-releasing paraventricular nucleus (PVH), the thermoregulatory medial preoptic area (MPO), and the melatonin-releasing pineal gland; (ii) SCN-independent pathways with light effects on mood through:

(a) alertness and sleep pathways: light, through melanopsin retinal ganglion cells, inhibits the VLPO promoting sleep neurons and activates numerous regions involved in alertness such as: monoaminergic areas (the locus coereleus, LC), thalamic regions (the paraventricular thalamus nucleus, PVT, and the intralaminar thalamic nucleus, ILT), hypothalamic regions (the dorsomedial hypothalamus/dorsal hypothalamic area, DMH/DHA, the lateral hypothalamus, LH, and the hypothalamus paraventricular nucleus, PVN).
(b) the orexinergic pathways: light induces excitatory responses in the serotoninergic dorsal raphe nucleus DRN by activating the orexinergic pathways.
(c) the monoamine (-serotonin, -dopamine)-dependent pathways.
(d) emotional processing pathways: light through ipRGCs activates limbic brain regions including the amygdala, the nucleus accumbens, the perihabenular nucleus, the left hippocampus. Light also activates the retina–ventral lateral geniculate nucleus and intergeniculate leaflet–lateral habenula pathway (retina–vLGN/IGL-LHb pathway) that regulates information from the limbic system to the midbrain monoaminergic centers.

Light effects on mood seem to be mainly associated with the activation of ipRGCs. Nevertheless, cone and rod contributions are less known but definitely have a role as emphasized in SAD and ERG studies for instance.

## 3. Result

### 3.1. Global Organization of Light Brain Effects

**Figure 2:**
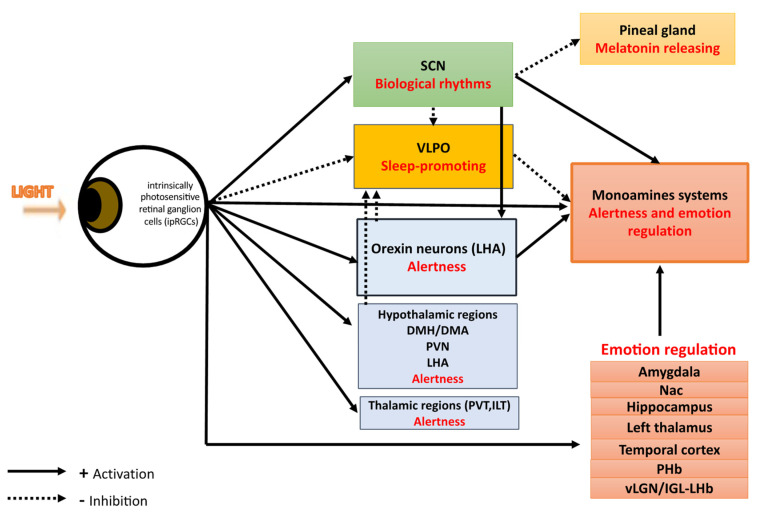
Photic regulation of mood and associated retina–brain pathways.

Light influences multiple systems governing physiology and behaviors [27]. These include sleep, alertness, emotions, cognition, body temperature, melatonin suppression, entrainment of circadian rhythms, regulation of heart, adrenal corticoid production, and mood [7,28,29,30,31,32]. Light detection occurs exclusively in the retina of mammals [33,34]. In addition to rod and cone photoreceptors, the third type of photoreceptors, ipRGCs, synthetizes the melanopsin photopigment specifically sensitive to luminance with a peak sensitivity to blue light (wavelengths 460–480 nm) [35,36,37]. Melanopsin relays information regarding light intensity to structures highly involved in the regulation of sleep, wakefulness, circadian rhythms, emotions, and mood. An important point to emphasize is that the mood regulation by light is absent in mice when ipRGCs are ablated [18,38], indicating that ipRGCs may be important to drive affective behavioral effects. Six subtypes of ipRGCs (M1-M6) have been described in rodents, four in humans [39,40,41] and their central projections differ largely, reflecting the numerous functions they participate in [33,42]. One of the major ipRGC targets is the master biological clock within hypothalamic suprachiasmatic nuclei (SCN), an endogenous central pacemaker that orchestrates all biological rhythms, such as circadian and seasonal rhythms [43]. This central pacemaker has an endogenous period of approximately 24.2 h for humans [44] and is temporally entrained to internal and external environmental synchronizers. Nevertheless, light appears as the most powerful environmental signal and entrain the biological clock to the daily period and to seasons [45,46,47]. Light projections of the SCN are multiple and include the sleep-inducing ventrolateral preoptic (VLPO) nucleus, structures involved in wake and appetite, such as the lateral hypothalamus LHA, areas involved in mood regulation, such as the dorsal raphe, the corticosteroid-releasing paraventricular nucleus (PVH), the thermoregulatory medial preoptic area (MPO), the melatonin-releasing pineal gland, and areas involved in mood regulation, such as the dorsal raphe [48,49]. Light exerts its biological effects by retinal SCN-independent pathways too (Figure 2). Indeed, IpRGCs project directly to several brain regions including sleep–wake areas such as hypothalamic areas with the VLPO and orexin neurons [7]. IpRGCs also project directly to the monoaminergic system that might ultimately contributed to light-dependent mood regulation, and to limbic regions such as the perihabenular nucleus (PHb) and the amygdala which are brain emotional areas, highlighting the direct regulation of mood by light [18]. Finally, light effects are also dependent on the light dose (determined by light irradiance level, duration of exposure, distance and angle from the light source), on light color spectrum, and on the time of day of light exposure [19,50,51] for which further research are warranted to determine the most efficient lighting parameters to use in the treatment of mood disorders [52] (Table 1). 

Light, via the ipRGCs, could exert mood regulation though a SCN-dependent pathway. SCN perceive and encode light information, then synchronize biological rhythms and simultaneously inhibit both the sleep-inducing ventrolateral preoptic (VLPO) nucleus and the melatonin-releasing pineal gland and activate structures involved in wake such as orexin neurons in the lateral hypothalamus (LHA), and areas directly involved in mood regulation—such as the dorsal raphe of the monoamines systems. Light, via the ipRGCs, could exert mood regulation though alertness effects by simultaneously inhibiting the sleep-inducing VLPO nuclei and activating diverse brain structures implicated in mood and alertness such as (i) the monoaminergic emotional and arousal system implicated in mood, (ii) the orexin neurons in the lateral hypothalamus (LHA), and the dorsomedial hypothalamus/dorsal hypothalamic area (DMH/DHA) which are both known to provide inhibitory control of the VLPO and to be excited during periods of wakefulness; (iii) the hypothalamus paraventricular (PVN), dorsomedial, and lateral hypothalamus (LHA) that are active during wakefulness; (iv) the thalamus, including the paraventricular thalamus nucleus (PVT) and the intralaminar thalamic nucleus (ILT) both independently associated with enhanced alertness and vigilance. In addition, light affects mood through the modulation of brain areas involved in emotional processing such as the amygdala, the nucleus accumbens (Nac), the left hippocampus, the left thalamus, the brainstem locus coeruleus, the temporal cortex, the perihabenular nucleus (PHb) and the lateral habenula (LHb).

### 3.2. Light Affects Mood through Different Retina–Brain Pathways

#### 3.2.1. Light Affects Mood through SCN-Dependent Pathway

The relationship between light and mood is known since antiquity. At the beginning of the 1980s, several key neuroscience discoveries were made regarding biological rhythms. First, researchers found that the SCN perceives and encodes changes in day length (i.e., photoperiod) and drives seasonal changes in downstream pathways and structures that mediate seasonal adaptations such as the duration of the nocturnal melatonin secretion [53]. Then, Wehr et al. in 1979 suggested that phase shifts of circadian rhythms could have an antidepressant effect [2] and, during the same period, Lewy et al. reported that light suppresses melatonin production [3], which provided a tool for manipulating circadian timing as well as seasonal behaviors [3]. These findings led clinical researchers to explore whether light could have an antidepressant effect by correcting the biological rhythm disturbances associated with depression. This light effect on mood via the circadian system in humans was really first illustrated with Kripke and colleagues in bipolar depression [4,5] then with Lewy and colleagues in seasonal depression [6], showing that the antidepressant effects of LT administered during early morning might be associated with a phase advance or an alignment of circadian rhythms. Then, Norman Rosenthal observed on himself that he was feeling depressed at the arrival of autumn and felt better in early spring. In 1984, with the help of his group of researchers at the National Institute of Mental Health in the United States, he published the first scientific description of the seasonal affective disorder (SAD) [10]. He reported that most patients experience depressive symptoms during autumn and winter probably due to the reduction of natural daylight in the environment, with full remission to euthymia or switch into (hypo) mania during spring and summer [10]. In order to correct this “light deficiency”, he decided to treat SAD with a simulation of summer daylength and thus published the first case series describing the antidepressant effect of light [10]. Since Rosenthal case series, SAD has been extensively explored and is now defined as a major depressive disorder that occurs annually in the autumn and winter due to the reduction of natural daylight affecting approximately 1 to 11.7% of the general population depending on gender, age and latitude [54,55,56]. It is now well established that SAD is in part caused by internal clock disturbances that lead to an internal misalignment of the circadian system—more frequently a phase delay of circadian rhythms—a diminished circadian amplitude during winter or an abnormal sensitivity to the short duration of daylight. These clock disturbances may relate in part to a decreased sensitivity of the circadian oscillators in the SCN to light. Therefore, it has been hypothesized that light therapy improves mood in SAD by resetting the circadian rhythms that were desynchronized due to the seasonal light deficiency [57]. Over the years, several trials and meta-analyses have extended the use of light to non-seasonal depression and demonstrated that light therapy can be proposed a first-line monotherapy not only for treating seasonal depression, but also non-seasonal depression [13,58,59,60], both for unipolar disorders [11,13,59,61] and BD [12,61,62,63,64]. These discoveries of the efficacy of light on mood disorder are not surprising, light is the most salient cue for entraining circadian rhythms [65] and a large number of studies demonstrated the involvement of biological rhythm abnormalities, both circadian and infradian ones, in seasonal and non-seasonal unipolar and bipolar disorder [19,66,67,68,69]. Indeed, regarding unipolar depressive disorders, the first hypothesis is linked to the phase advance of circadian rhythms (phase advance of the sleep period, phase advance of core body temperature and cortisol for instance) involving clock genes variants [70]. It is thus hypothesized that this misalignment between circadian rhythms and the light–dark cycle would lead to depressive symptoms and so would explain both the early awakening and the shortening of the rapid eye movement (REM) sleep latency, two circadian markers frequently found altered in depressive disorders [71]. Regarding bipolar disorders (BD), several works demonstrate also the involvement of biological rhythm alterations, both circadian and seasonal ones [72]. These abnormalities in chronobiological rhythms appear during all phases of the illness, during acute phase or during remission, and include changes in mood, appetite, energy, and abnormalities in sleep–wake rhythms [72]. The highlighted abnormalities of the sleep–wake rhythm, are mainly a vesperal chronotype, a more languid and rigid circadian typology, a longer sleep duration, a longer sleep latency, a poorer sleep efficiency, a more frequent awakening after the first sleep, a poorer inter-daily stability, a variability in the bedtime, the time of awakening, or in the sleep duration and efficiency [73,74]. Hormonally, the chronobiological abnormalities found are mainly a phase delay and decreased melatonin secretion, a melatoninergic hypersensitivity to light, elevated cortisol levels at night, and an early nadir on the temperature curves [75,76,77,78,79]. Finally, genotype-mood disorder phenotype correlations have been made with core clock genes and melatonin synthesis pathway genes, including a variant of the ASMT gene promoter associated with poorer quality sleep, a TIMELESS variant associated with languid rhythms (i.e., sleep inertia) as well as a more vesperal profile and a RORA variant with less flexible profile of life rhythms [80,81,82].

To summarize, SCN-dependent pathways of mood regulation begin with the retinal-SCN neural track activated by the ipRGCs synthetizing the melanopsin photopigment in response to light (Figure 1). Then, SCN perceive and encode light information and (i) synchronize all biological rhythms, such as circadian and seasonal rhythms [43] and (ii) project light information to many areas including the sleep-inducing ventrolateral preoptic (VLPO) nucleus, the structures involved in wake, appetite, such as the lateral hypothalamus LHA, areas directly involved in mood regulation such as the dorsal raphe, and to others areas such as the corticosteroid-releasing paraventricular nucleus (PVH), the thermoregulatory medial preoptic area (MPO), and the melatonin-releasing pineal gland [48,49] (Figure 1 and Figure 2).

#### 3.2.2. Light Affects Mood through SCN-Independent Pathway

Until recently, effects of light on mood and its antidepressant effects have been primarily attributed to light’s effect on the biological clock. Nevertheless, light may also influence mood through distinct SCN-independent pathways more recently evidenced and summarized in this section (Figure 1 and Figure 2).

##### Light Effects on Mood through Sleep and Alertness Pathways

1. Light Effects on Mood through the Modulation of the Sleep Homeostasis

The influence of light on mood through the sleep homeostasis is well illustrated in SAD. Several studies comparing healthy patients and patients with SAD indicate that the homeostatic “process S” is directly modulated by light. First, patients with SAD compared with healthy controls show differences in sleep duration during Winter [83,84]. Secondly, studies have shown significant differences in sleep architecture between patients with SAD and healthy controls in winter compared to summer: patients with SAD, compared with healthy controls, have lower delta activity or slow-wave sleep (SWS) and lower sleep efficiency in winter compared to summer, suggesting that photic or photoperiodic information play a key role in sleep homeostasis in SAD [7,83]. Thirdly, in SAD patients, LT improves sleep efficiency and delta sleep [84,85]. This positive effect of light on delta activity is similarly observed after administration of either morning or evening light, suggesting that light impacts the delta power spectrum and that positive effect does not result from modulation of the biological clock [84,85]. Light’s impact on EEG delta activity may so rather result from SCN-independent effects of light. In addition, some studies suggest that this light effect on delta activity could be melanopsin-dependent and may implicate the VLPO sleep-regulatory structure. Indeed, in patients with SAD, it has been observed that increases in EEG delta activity depends on the spectral component of light with blue light being more effective than red light in decreasing depressive symptomatology [86,87]. In mice the absence of melanopsin affect light effects on sleep and the electrocorticogram activity, and light through melanopsin retinal ganglion cells activates the VLPO [86,87]. Taken together, these results emphasize the close relationship between seasonal changes of sunshine, daylength, sleep need and mood. These results are also suggesting the hypothesis of direct effects of light on the VLPO through activation of ipRGCs (Figure 1). Further studies using animal models, healthy volunteers and patients affected with seasonal depression are needed in order to understand the complex relationships between light, sleep homeostasis and mood (Table 1). Nevertheless, clinical studies have already shown that sleep deprivation, which increases the sleep pressure, has a rapid-acting antidepressant effect [88]. Interestingly, this clinical efficacy is improved by adding other chronotherapeutic approaches, such as LT [89].

2. Light Affects Mood through the Modulation of Alertness

Lack of alertness or excessive daytime sleepiness (EDS) is a complaint frequently met in all affective disorders such as seasonal affective disorder but also in non-seasonal major depressive disorder and non-seasonal bipolar disorder [90]. EDS impairs concentration, memory and mood, which further negatively impact global functioning and wellbeing, and may be associated with suicidal ideation in patients with MDD [91]. Light is known to exert an acute alerting action in humans. This alerting response to light in humans depends on factors such as the illuminance levels, exposure duration, timing and wavelength of light [7,29,92]. Neuroanatomical and neurophysiological findings have shown that, in addition to its powerful alertness influence through the SCN [7,93], light may enhance alertness through direct mechanisms activating alertness centers in the brain [29,94,95]. Badia et al. were among the first to explore how light modulates alertness by applying a 90 min bright light therapy (BLT) of 5000 lux or a 90 min dim light (DL) of 50 lux during the subjective night [29]. They showed that alertness measured by EEG beta activity (i.e., a rapid waked EEG activity) was immediately increased during BLT session. Since Badia et al., it has been discovered that light could exert its alerting effect by simultaneously inhibiting the sleep-inducing VLPO nuclei and activating the monoaminergic arousal system implicated in mood [87]. These effects are attenuated and delayed in the absence of melanopsin, demonstrating that light would directly exert this alerting effect via the ipRGCs [87]. In addition, more recently Milosavljevic et al. observed that in mice the activation of ipRGCs not only increased alertness and arousal, but also had immediate effects on diverse brain structures implicated in mood, attention and alertness [96]. These brain structures included: (i) the dorsomedial hypothalamus/dorsal hypothalamic area (DMH/DHA); DMH/DHA is known to provide inhibitory control of the VLPO [96] and is known to be excited during periods of wakefulness [48,97]; (ii) the hypothalamus paraventricular, dorsomedial, and lateral hypothalamus that are active during wakefulness; (iii) the thalamus, including the paraventricular thalamus nucleus (PVT) and the intralaminar thalamic nucleus (ILT) both independently associated with enhanced alertness and vigilance [98,99]; and (iv) the limbic system including the amygdala and the nucleus accumbens. The amygdala has been reported to be light activated in humans [100] and also to receive direct inputs from ipRGCs [33]. The nucleus accumbens is a region associated with enhanced motivation and reward-seeking behavior [101]. Finally, in humans, several studies using positron emission tomography (PET)119 or functional magnetic resonance imaging (fMRI), observed that light also induced changes in multiple brain areas involved on both alertness and mood such as the thalamus (with activations linearly correlated with the improvement of alertness) and such as the locus coeruleus (LC) [30,102]. At the same time, light inhibited brain areas promoting sleep such as the VLPO [103]. All these discoveries demonstrated that light could thus enhance mood by increasing alertness with several direct tracts from ipRGCs to wake and mood areas (Figure 1 and Figure 2). These findings pave the way for further research to better unravel the relationships between light, mood and alertness (Table 1). Among wakefulness pathways, the orexinergic system, plays a key role in modulating arousal, but also plays a key role in many other functions including mood. In this context, the following paragraph will synthetize new insights about the relationships between light, mood and the orexinergic pathways.

##### Light Affects Mood via the Retina–Orexinergic Pathways

Orexin neurons play a key role in the regulation of appetite, affect, and reward system, as well as in the regulation of the sleep–wake cycle [104]. Orexin neurons activate wake promoting nuclei, by releasing norepinephrine via the Locus Coereleus (LC) [105]. In contrast, the orexin inhibition facilitates sleep initiation and maintenance [106]. In addition, orexin neurons have been postulated to have a role in depressive mood with evidence of abnormalities of their activity in both animal models of depression and in humans with MDD [107,108]. Indeed, it has been observed from pre-clinical studies a positive correlation between the amygdala orexin receptor expression and the manifestation of depressive behaviors [107]. Moreover, severe depressive behaviors were associated with decreased hippocampal orexin expression in the amygdala [108]. In humans, abnormalities in the activity of orexin neurons have been also observed in MDD. For instance, one study evaluating cerebrospinal fluid levels of the A orexin (i.e., which is one of the two subtypes of orexin) found a decrease in the circadian amplitude variation in patients with MDD compared with healthy controls [109]. Orexin neurons also mediate the mood effects of light [107,108] by receiving direct retinal inputs [33] and indirect retinal inputs from other hypothalamic nuclei including the SCN [110,111]. Orexin neurons innervate the dorsal raphe nuclei (DRN) implicated in mood regulation, as reported in nocturnal rats [112] and also in diurnal rats [113]. A study from Adidharma et al. demonstrated the role of orexin neurons in mediating the effects of light on the mood-regulating monoaminergic areas [114]. They showed that orexin neurons are part of the tract through which light information is transmitted to the serotonergic nucleus within the dorsal raphe (DRN) [115]. They observed that rats housed in winter-like lighting conditions then presented depression-like behaviors, supporting their potential use as an animal model of SAD [115,116,117,118]. Then, to better explore the neural pathways mediating the effects of light on mood, an acute light pulse was administered in the early subjective day to rats housed in constant darkness. This light pulse was intended to mimic the acute effect of light therapy, which is generally carried out during early daytime [119,120]. Using *c-Fos* marker, the brain responses were examined, in three interconnected brain regions/cell populations that could potentially be involved in mediating the effects of light on mood regulation: the principal circadian clock in the SCN [121], the wakefulness-promoting orexin neurons [106], and the largest serotonergic nucleus within the dorsal raphe (DRN) [115,122]. Their study showed first that light exposure during subjective daytime increases immediately neural activity in orexinergic cells and in the DRN but not in the SCN [114]. Furthermore, blocking the orexinergic signaling using an orexin receptor-1 (OXR1) antagonist inhibits the light-induced neural activity in the DRN suggesting that light induces excitatory responses in the serotoninergic DRN through activating orexinergic pathways. Thus, their results support the idea that the activation of the wakefulness promoting orexinergic system underlies the therapeutically effects of light on SAD. Their study focused on the DRN, but orexin neurons project to and directly regulate all monoaminergic neurons that are involved in mood regulation [123]. Therefore, the orexinergic pathway seems to be very a key player to mediate the effects of light on monoaminergic system that ultimately contribute to light-dependent mood regulation (Figure 1). Therefore, in the following section, a closer look on the effects of light on monoaminergic systems is proposed.

##### Light May Affect Mood through the Monoamine-Dependent Pathways

1. Light Effects on the Serotonin-Dependent Pathways

It has been well established that light influences serotonin (5-HT) turnover: the rate of production of serotonin by the brain is the lowest in winter and rapidly increases with longer daylength during summer [124]. Light also influences serotonin transporter binding levels in the brain, which increase in autumn and winter compared to spring and summer [125].

The serotonergic system and the influence of light on the serotonergic system have been extensively investigated in all mood disorders, including SAD, unipolar depression and BD [126]. In addition, it has been reported higher seasonal serotonin transporter (5-HTT) fluctuations in SAD, when compared to healthy controls, in all brain regions, including mood regulation areas such as the anterior cingulate (ACC) and prefrontal cortex (PFC). It has been also observed that 5-HTT density and affinity were significantly decreased in the ACC and PFC after 2 weeks of LT and correlated with the decrease of depressive symptoms [127]. All these findings suggest that (i) in SAD, light impacts seasonal fluctuations of 5-HT and 5-HTT levels, and that (ii) 5-HTT plays a central role in the direct mechanisms of the antidepressant effect of light [75]. In other words, increasing central 5-HT availability seems to be an important effect of LT in SAD. How LT acts to modulate 5HTT affinity for serotonin is however still unclear. Furthermore, serotonin has a strong and bidirectional relationship with the biological clock [128]. Indeed, serotonin modulates the SCN response to light [129], and light induces indirect signaling, from the SCN to the raphe median nucleus [73]. Another possible hypothesis of the antidepressant effect of light through the modulation of 5-HTT levels is that light may induce direct signaling (i.e., independently of the SCN) between the retina and the serotoninergic system such as a direct action on the raphe dorsal nucleus (Figure 1 and Figure 2).

2. Light Effects on the Dopamine-Dependent Pathways

Dopamine also regulates sleep–wake rhythms [130] and is known to contribute to the adaptation to the light/dark cycles in retina photoreceptors [131]. Dopamine may also play a role in the pathophysiology of SAD. In SAD, retinal alterations such as a lower rod retinal sensitivity and lower cone maximal amplitude assessed by the electroretinogram (ERG) are apparent during the depressive phase in autumn/winter, and are normalized after 4 weeks of LT or during the summer [132]. These findings suggest that these ERG changes regarding cone and rod functions are state markers for SAD [132]. These results raise questions about the underlying roles of the dopamine in SAD since multiple dopamine-dependent physiological mechanisms act through cone and rod circuits [131]. Other studies have also suggested the contribution of the cerebral dopaminergic system in SAD, including the decreased dopamine transporter (DAT) availability observed in untreated symptomatic depressed SAD patients [133]. LT appears to stimulate the dopamine production in the retina [134]. In this later work, authors suggest that the winter-induced retinal dopamine deficiency may cause SAD and that light reverses this syndrome. Finally, a recent preclinical study indicates that variations in the levels of hypothalamic DAT may act to model seasonal changes in a BD model [135] and suggest that light may influence DAT binding levels in the brain. Actually, their data reveal the potential contribution of the photoperiod-induced neuroplasticity within an identified circuit of the hypothalamus, linked with reduced DAT function, underlying switching between states in BD. Mice with reduced DAT expression exhibited hypersensitivity to summer-like and winter-like photoperiods, including more extreme mania-relevant and depression relevant behaviors [135]. Further clinical studies are warranted in the future to confirm if DAT could be considered as an important central biomarker of SAD and/or seasonal BD, and could thus play a role in the direct and SCN-independent mechanisms of light (Figure 1).

##### Light Affects Mood through Emotional Processing Pathways

1. Light Effects on Brain Areas Involved in Emotional Processing

Mood disorders such as MDD or BD are characterized by altered structural or functional changes in areas involved in emotional processing, such as the amygdala, the hypothalamus, and the hippocampus [136]. Responses to emotional stimuli can have a large impact on mood [136]. LT is an effective treatment for mood disorders and so several authors assessed whether light affect mood through the modulation of brain areas involved in emotional processing. A first study of Vandewalle et al. assessed whether light can acutely influence normal brain emotional processing [100]. Their study showed that amygdala was one of the emotional brain area acutely affected by changes in ambient light [94]. This is not surprising as amygdala is known to be a core component of the emotional brain [136,137] that receives sparse direct projections from ipRGCs [33]. They also showed that blue light could induce the activation of three others cores components of the emotional brain: the left hippocampus, left thalamus and the brainstem locus coeruleus. The same team then explored whether light could directly modulate emotional brain responses to emotional stimuli in healthy subject by using functional MRI (fMRI) and a validated auditory emotional task [100]. They observed that blue light increased responses to emotional stimuli in the voice area of the temporal cortex and in the hippocampus, two other areas both implicated in emotional processing [100]. They also observed during blue illumination that emotional processing was associated with an enhanced functional coupling between the voice areas, amygdala and hypothalamus [100]. Their findings suggest that ambient blue light strengthens emotional brain reactivity and might promote accurate and contrasting responses to emotional signals, which could enhance efficient mood regulation processes. These results have important implications for the understanding of the mechanisms by which changes in lighting environment enhance mood [28,119]. More recently, Milosavljevic et al. demonstrated in mice that light has immediate effects on diverse brain structures implicated in mood, including emotional processing areas, such as the amygdala and the nucleus accumbens [95]. They also highlighted the key role of melanopsin-expressing retinal ganglion cells to emotional brain area responses to light. Finally, two new retino–brain pathways implicated also in emotion regulation has been recently discovered and will be detailed in the two following sections. These findings taken together confirmed that light might induce mood changes the refereeeehrough emotional processing pathways [9,138] (Figure 1 and Figure 2).

2. Light Effects on the Retino–Perihabenular Nucleus Pathway

A key recent work reported that another SCN-independent pathway might mediate light-induced mood changes through emotional processing pathways [138]. In this study, Fernandez et al., found that a subset of ipRGCs (predominantly M1) in mice directly innervated the perihabenular nucleus (PHb) a region of the dorsal thalamus, known to be implicated in emotion regulation. They interestingly showed that an irregular light stimulation led to depressive-like behaviors associated with changes in the thalamic PHb. PHb neurons show a robust rhythmic expression of the clock gene PER2 under normal light conditions. In this study, when mice were housed under the T7 cycle (i.e., 3.5 h of light followed by 3.5 h of dark), the PER2 rhythmicity was abolished in the PHb [139,140]. Thus, these findings revealed that irregular light/dark cycles perturb the biological clock in the PHb through an ipRGCs-dependent circuit, then inducing depressive behaviors. The PHb is integrated into a loop of limbic thalamic nuclei and is connected to thalamo–frontocortical loop [141]: PHb neurons project to several mood regulating centers with collateral projections to the ventro-median prefrontal vortex (vmPFC), the dorsal and ventral striatum. The prefrontal cortex (PFC) is known to be fundamental to mood regulation and has been consistently implicated in MDD by imaging studies of patients [142,143,144] and using animal models of mood disorders [145]. The dorsomedial striatum participates to a thalamo–frontocortical loop, which is involved in affective emotional processing. The ventral striatum, the third target of PHb neurons, and mostly the nucleus accumbens (NAc) included in the ventral striatum, has been extensively implicated in mood and depression [146]. Furthermore, the study of Fernandez et al. demonstrated that the PHb is both necessary and sufficient for driving the effects of light on affective behavior. Indeed, they reported that inhibiting PHb neurons blocked the mood alterations triggered by abnormal light cycles and they reported that chronic activation exclusively of projecting PHb neurons induced significant alterations in mood. Chronic exposure to light at night in humans may cause similar neuronal changes leading to mood alterations.

3. Light Effects on the Retina vLGN/IGL-LHb Pathway

Interestingly, Huang et al. recently observed that light influences also depressive-like behaviors in mice through a pathway linking the retina and the lateral habenula (LHb) [9]. The LHb from the epithalamus regulates information from the limbic system to the midbrain monoaminergic centers [147]. Stress factors can activate the LHb and induce depressive-like behaviors [147,148,149,150,151], whereas the inhibition of the LHb improves depressive symptoms [152]. In addition, recent pre-clinical studies have suggested that light can influence neuronal activity in the LHb, leading to depressive-like behaviors [38,153]. Indeed, in the Huang et al. study, they identified a retinal-brain pathway connecting the retina and LHb in the mouse [9]. In the retina, a subset of M4-type retinal ganglion cells (RGCs) innervates GABA neurons in the ventral lateral geniculate nucleus and intergeniculate leaflet (vLGN/IGL) [9], which in turn inhibit the neural activity of the LHb. They demonstrated that the activation of the retina–vLGN/IGL-LHb pathway is required for the antidepressant effects of BLT. Moreover, these works also showed that either the inhibition of LHb-projecting vLGN/IGL neurons or the activation of LHb postsynaptic neurons abolish the antidepressant effects of LT [9]. These works reported an absolutely new anatomically distinct neuronal circuit involved in light mediated mood deficits.

## 4. Conclusions

Recent findings show the existence of direct neural tracts and pathways from photoreceptors to brain regions directly or indirectly involved in mood regulation (Figure 1). Unveiling the structure and function of these neural circuits related to the retina and response to light are crucial to better understand how light exerts its influence on mood. Further researches are needed to detail how these mechanisms interplay, what could be their possible functional independency, and how they contribute to the photic regulation of mood (Table 1).

## Figures and Tables

**Table 1:** What is known and what is still unknown.

	What Is Known?	What Is Still Unknown?
Retina brain pathways of mood regulation by light	Light affects mood through different retina–brain pathways: Light affects mood through SCN-dependent pathway Light affects mood through SCN-independent pathways **In the SCN-dependent pathway**, the SCN perceive and encode light information, then synchronize biological rhythms and project light information to many areas including: the sleep-inducing ventrolateral preoptic (VLPO) nucleus, structures involved in wake and appetite—such as the lateral hypothalamus LHA-, areas directly involved in mood regulation—such as the dorsal raphe-, the corticosteroid-releasing paraventricular nucleus (PVH), the thermoregulatory medial preoptic area (MPO), and the melatonin-releasing pineal gland; **In the SCN-independent pathways,** light affects mood through: (a) alertness and sleep pathways: light, through melanopsin retinal ganglion cells, inhibits the VLPO promoting sleep neurons and activates numerous regions involved in alertness such as: monoaminergic areas (the locus coereleus), thalamic regions, hypothalamic regions. (b) the orexinergic pathways: light induces excitatory responses in the serotoninergic dorsal raphe nucleus (DRN) by activating the orexinergic pathways. (c) the monoamine (-serotonin, -dopamine)-dependent pathways. (d) emotional processing pathways: light through ipRGCs activates limbic brain regions including the amygdala, the nucleus accumbens, the perihabenular nucleus, the left hippocampus. Light also activates the retina–ventral lateral geniculate nucleus and intergeniculate leaflet–lateral habenula pathway (retina–vLGN/IGL–LHb pathway) that regulates information from the limbic system to the midbrain monoaminergic centers.	All neural tracts and pathways from photoreceptors to brain regions directly or indirectly involved in mood regulation are not known. For instance, four (M1–M4) subtypes of intrinsically photosensitive retinal ganglion cells (ipRGCs) have been identified in humans and 6 in rodents. M4-type (ipRGCs) can directly innervate LHb-projecting vLGN/IGL neurons. M1- type (ipRGCs) directly innervates the perihabenular nucleus (PHb). This finding suggests that there are different subtypes of ipRGCs that participate in different pathway of light effect on mood. Further studies are needed to precise the subtype involved in each pathway. Light effects on mood seem to be mainly associated with the activation of ipRGCs. Nevertheless, cone and rod contributions are less known but have definitely a role as emphasized in SAD and ERG studies for instance. Furthers research are warranted to determine precise light projections, and pathways involved in mood regulation.
Light physiological effects contributing to mood	The SCN-dependent and the SCN-independent pathways seem to target the three main mechanisms of light effect on mood: sleep homeostasis, alertness enhancement, and emotion regulation by possibly activating same regions such as VLPO, monoamine pathways, orexin neurons.	How each pathway contributes to the photic regulation of mood? SCN-dependent and SCN-independent pathways target possibly the same mechanisms, but their differences in terms of effects are unknown. How all these different retina–brain pathways interplay? What could be their possible independent functioning? Do the SCN-dependent and/or the SCN-independent pathway provide a time-of-day effect while the other reacts to a threshold, or vice-versa, or both? Further studies using animal models, healthy volunteers and patients affected with seasonal depression and mood disorder are needed in order to better understand the complex relationships between light, mood, sleep homeostasis, alertness and emotion regulation.
Parameters influencing light mood effects	Light effects are dependent of light dose, determined by: - light irradiance level, - duration of exposure, - distance from the light source, - angle from the light source. light color spectrum time of day of light exposure	Further researches are warranted to determine the most efficient lighting parameters to use in the treatment of mood disorders and its different subtypes.

## Data Availability

Not applicable.
